# APEX1 predicts poor prognosis of gallbladder cancer and affects biological properties of CD133^+^ GBC-SD cells via upregulating Jagged1

**DOI:** 10.7150/jca.83356

**Published:** 2023-05-15

**Authors:** Zhengchun Wu, Ziru Liu, Yi Sun, Yuan Yuan, Qiong Zou, Yun Wen, Jia Luo, Rushi Liu

**Affiliations:** 1 Department of Hepatobiliary and Intestinal Surgery, Hunan Cancer Hospital and the Affiliated Cancer Hospital of Xiangya School of Medicine, Central South University, Changsha, Hunan410013, China.; 2 Department of General Surgery, Second Xiangya Hospital, Central South University, Changsha, Hunan410011, China.; 3 Department of Pathology, Second Xiangya Hospital, Central South University, Changsha, Hunan410011, China.; 4 Department of Pathology, Third Xiangya Hospital, Central South University, Changsha, Hunan410013, China.; 5 Laboratory of Medical Molecular and Immunological Diagnostics, School of medicine, Hunan Normal University, Changsha, Hunan 410013, China.

**Keywords:** Gallbladder cancer, APEX1, Jagged1, Prognosis, CD133^+^ cancer cells

## Abstract

Although APEX1 is associated with the tumorigenesis and progression of some human cancer types, the function of APEX1 in gallbladder cancer (GBC) is unclear. In this study, we found that APEX1 expression is up-regulated in GBC tissues, and APEX1 positive expression is related to aggressive clinicopathological features and poor prognosis of GBC. APEX1 was an independent risk factor of GBC prognosis, and presented some pathological diagnostic significance in GBC. Furthermore, APEX1 was overexpressed in CD133^+^ GBC-SD cells in comparison with GBC-SD cells. APEX1 knockdown increased the sensitivity of CD133^+^ GBC-SD cells to 5-Fluorouracil via facilitating cell necrosis and apoptosis. APEX1 knockdown in CD133^+^ GBC-SD cells dramatically inhibited cell proliferation, migration, and invasion, and promoted cell apoptosis *in vitro*. APEX1 knockdown in CD133^+^ GBC-SD cells accelerated tumor growth in the xenograft models. Mechanistically, APEX1 affected these malignant properties via upregulating Jagged1 in CD133^+^ GBC-SD cells. Thus, APEX1 is a promising prognostic biomarker, and a potential therapeutic target for GBC.

## Introduction

Gallbladder cancer (GBC) is the most malignant tumor of the biliary tract system, with poor prognosis and high mortality. GBC is mainly comprised of adenocarcinoma (AC) and squamous cell/adenosquamous carcinoma (SC/ASC) 1, 2. Since lacking special clinical signs and symptoms in the early stage, GBC is usually diagnosed in the late stage with serosal invasion and metastasis to other organs. GBC early occurs lymphatic metastasis, nerve metastasis and hemorrhagic metastasis which reduces the overall survival rate of GBC patients 3, 4. Currently, radical resection remains the only strategy to cure GBC. However, most GBC patients have missed the opportunity to receive radical surgery when they are diagnosed, and can merely chose adjuvant therapy such as chemotherapy and radiotherapy which can't obtain satisfactory curative effect 5-7. Thus, enhancing the early diagnosis rate and elucidating the mechanism of metastasis and drug resistance of GBC are very crucial for improving the prognosis and finding new therapeutic targets for GBC.

CD133, a transmembrane glycoprotein encoded by the PROM1 gene, is originally identified in human hematopoietic stem cells and progenitor cells 8. As an important stem cell marker, CD133 is expressed in embryonic stem cells, somatic stem cells and circulating endothelial progenitor cells 9. With some stem cell properties, CD133^+^ cell is able to self-renew, proliferate and differentiate into different cell types 9. Recently, CD133 has been viewed as a reliable biomarker of cancer stem cells, and is expressed in several cancer stem cell types 10-12. CD133^+^ cancer cells are closely related to the occurrence, development, metastasis and recurrence of tumors 13, 14. Additionally, CD133^+^ cancer cells present highly resistant to conventional chemoradiotherapy, which may be owed to the strong ability of CD133^+^ tumor cells to repair DNA damage. Multiple CD133^+^ cancer cells show significantly enhanced DNA repair capacity in glioma cells, medulloblastoma cells, prostate cancer cells and lung cancer cells 15-18. Compared with CD133^-^ GBC cells, CD133^+^ GBC cells demonstrated an increased potential for tumor formation, cell proliferation, invasion and resistance to chemotherapeutic agents 19-21, indicating that CD133^+^ cancer cell plays an important role in GBC tumorigenesis and progression. Hence, studying the role of DNA repair genes in CD133^+^ GBC cells may be of great significance for finding new therapeutic targets and improving the prognosis of GBC.

Apurinic-apyrimidinic endonuclease-1 (APEX1), a multifunctional protein, is consisted of 318 amino acids, with two functional domains. As a key rate-limiting enzyme in the DNA base excision repair (BER) pathway, APEX1 can remove and repair the apurinic/apyrimidinic site of damaged DNA 22, 23. Additionally, APEX1 with redox activity is capable of modulating the binding of various transcription factors to DNA, such as EGR1, NF-κB, P53, and HIF-1α 24. APEX1 participates in the occurrence and progression of tumors, cardiovascular diseases and neurodegenerative diseases 25. APEX1 is involved in regulating various tumor biological characteristics including cell cycle, proliferation, migration, invasion, apoptosis, angiogenesis, epithelial-mesenchymal transition (EMT), and sensitivity to chemoradiotherapy 24, 26, 27. APEX1 expression presents up-regulated in several human cancer types, which is correlated to aggressive clinicopathological parameter and poor prognosis of these cancer 23, 28-30. Moreover, APEX1 functions an important role in cancer stem cell self- renewal, differentiation, death, and survival, and can facilitate cancer stem cells resist to chemoradiotherapy 27, 31. However, the biological role of APEX1 in GBC is never reported.

In this study, we investigated the expression and clinicopathological significance of APEX1 in GBC, and clarified the biological function of APEX1 in CD133^+^ GBC cells.

## Material and Methods

### Case selection

This study included 69 SC/ASC patients and 146 AC patients. The detailed criteria for patient inclusion were that the enrolled patients were histologically diagnosed by two different pathologists, and never received chemotherapy or radiation therapy preoperatively and postoperatively. The detailed criteria for patient exclusion were that the patients received chemotherapy or radiation therapy were excluded. The SC/ASC samples were obtained from January 2001 to December 2013, including 16 from Xiangya Hospital, 31 from Second Xiangya Hospital, 10 from Third Xiangya Hospital, 5 from Hunan Provincial People Hospital, 5 from Hunan Cancer Hospital, 1 from Changde Central Hospital, and 1 from Loudi Central Hospital. The AC samples were collected at Second Xiangya Hospital and Third Xiangya Hospital from January 2008 and December 2013. The diagnosis of GBC subtypes was based on the recommendations of the 7th American Joint Committee on Cancer. We followed patients by letters or telephone for 2 years to collect survival information, and the patients with survival time over 2 years were defined as censored cases. This study obtained the approval of the Ethics Committee of Central South University, and was conducted based on the Declaration of Helsinki. Informed consents were obtained from all participants included in this study.

### EnVision Immunohistochemistry

EnVision^TM^ Detection Kit (Dako Laboratories, CA, USA) was applied for tissues immunohistochemical staining which was conducted as previous description 32. Briefly, paraffin-embedded tissues were cut to 4-μm thick sections and were then deparaffinized, followed by peroxidase inhibitor (3% H_2_O_2_) incubation in the dark for 15 min, and EDTA-trypsin digestion for 15 min. After the rabbit anti-human APEX1 incubation (Santa Cruz Biotechnology, CA, USA) for 1 h at 37 ℃, the sections were soaked with PBS for 3 × 5 min and were then incubated with the HRP-conjugated anti-rabbit second antibody (Santa Cruz Biotechnology, CA, USA) for 0.5 h at 37℃. Then, DAB staining and hematoxylin counter-staining were performed sequentially on the sections. The sections were dehydrated in graded ethanol (70%-100%), soaked in xylene, and mounted with neutral balsam.

Two different pathologists observed independently ten random fields per section, and the percent of positive stained cells was counted. Strength of staining was classified on a scale of 1 to 3, with 1 denotes no positive staining or uncertainly weak staining, 2 denotes weak to moderate staining, and 3 denotes moderate to strong staining. A section with ≥10% positive stained cells and staining strength ≥2 was viewed as positive expression. The few sections with 5%-10% positive stained cells and staining strength ˃2 were also determined as positive staining.

### Western Blot

Total protein extraction was performed in tissues or cell samples, and total protein concentrations were determined. After being separated on SDS-PAGE gel, proteins were transferred to PVDF membrane (Bio-Rad) which was then blocked with 5% skimmed milk. Next, the membrane was incubated with primary antibody at 4℃ overnight and then with second antibody (Proteintech) for 1 h at room temperature. The antigen-antibody complexes were tested with ECL reagent (Monad Biotech. Co.Ltd., China). The primary antibodies were listed as follows: APEX1 (Proteintech), β-actin (Proteintech), Jagged1 (Abcam), DLL4 (Proteintech), Notch1 (Abcam), Notch3 (Abcam), RBP-JK (Proteintech), Hes1 (Abcam), Hey1 (Proteintech).

### Real-Time Quantitative PCR (qRT-PCR)

Total RNA was extracted from tissues or cells with Trizol reagent (Beijing Dingguo Changsheng Biotech, Co., Ltd., China). cDNA was synthesized by RNA reverse-transcription using PrimeScript RT reagent Kit (Takara Biomedical Tech, Co., Ltd., China). SYBR Premix Ex Taq II (Takara Co., Ltd., China) was used to conduct qRT-PCR on the CFX connect system (Bio-Rad Co., Ltd., USA). This study selected GAPDH as an internal control. The primers were all synthesized from Tsingke Biological Technology Co., (Changsha, Hunan, China), and their sequences were showed as next:

GAPDH: Forward 5′-ATGACCACAGTCCATGCCATCA-3′,

Reverse 5′-TTACTCCTTGGAGGCCATGTAG-3′;

APEX1: Forward 5′-CCAGCCCTGTATGAGGACC-3′,

Reverse 5′-GGAGCTGACCAGTATTGATGAGA-3′;

Jagged1: Forward 5'-TGGATGAGATCAATGGCTACCG-3',

Reverse 5'-CTCGCTGTGCCCTTTGTGGA-3';

DLL4: Forward 5'-TTGCCACCAGATGCACTCAT-3',

Reverse 5'-CACATAGTGGCCGAAGTGGT-3';

Notch1: Forward 5'-GCCGCCTTTGTGCTTCTGTTCTTC-3',

Reverse 5'-TCTTGGTCTCCAGGTCCTCGTCCC-3';

Notch3: Forward 5'-GCCGTCAGTGGACTCAACACCAT-3',

Reverse 5'-ACGCACATTGACATCCATGCCAT-3'

RBP-JK: Forward 5'-TCTGCATTCCGAGAAGGTTG-3',

Reverse 5'-GGTAAAGGTAAGGCTGGTGG-3';

Hes1: Forward 5'-CGGACATTCTGGAAATGACAGTGAAGC-3',

Reverse 5'-CGCACCTCGGTATTAACGCCCTC-3';

Hey1: Forward 5'-TGCATACGGCAGGAGGGAAAG-3',

Reverse 5'-GTCGAACTCGAAGCGGGTCAG-3'.

### Cell Lines and Culture

The human gallbladder cancer cell line GBC-SD was purchased from the Cell Bank of the Chinese Academy of Sciences (Shanghai, China). The culture of cells was performed as previous described 33.

### Isolation of CD133^+^ cell Population

The CD133^+^ GBC-SD cells were isolated from GBC-SD cells by magnetic Cell Sorting. The detail procedure was presented in a previous study 33.

### Inhibition of APEX1 and Jagged1 expression and overexpression of Jagged1

The plasmids were purchased from GeneChem (Shanghai, China). This study used plasmids including APEX1 shRNA plasmid, Jagged1 shRNA plasmid, Jagged1 ectopic expression plasmid, and negative control plasmid. Transfection of the plasmids were conducted with Lipofectamine 2000 (Invitrogen, United States) in accordance with manufacturer's instructions.

### CCK8 assay and colony formation assay

Cell Counting Kit-8 (CCK8) (DOJINDO, Japan) was applied to determine cell proliferation. Cells were seeded at a density of 1 × 10^4^ cells per well in 96-well culture plates. Four wells of each group were detected every day. Each well was put CCK-8 solution and then incubated at 37℃ for 4 h. Finally, the absorbance was tested by application of a microplate reader. Colony formation assays were conducted in 6-well culture plates. Cells were seeded at a density of 4 × 10^2^ cells/well and then cultured for 2 weeks at 37°C. Finally, colonies per well were stained with 5% crystal violet and counted.

### Cell migration and invasion assay

Cell migration was detected in a 24-well transwell plate (Corning, United States). Cells were resuspended in serum-free medium, then 1 × 10^5^ cells were placed into the upper chamber, and the bottom well was added complete medium. After culture for 48 h at 37℃, the cells remained in the upper face of the filters were removed. The cells migrating to the lower face were fixed with 4% formaldehyde and stained with 0.5% crystal violet, and then the number of these cells was calculated under a microscope. The procedure of cell invasion assay was essentially similar to the migration assay, except for the membrane filters with Matrigel (Becton, Dickinson and Company, United States).

### Flow Cytometry Assay for Apoptosis

Cells were cultured in a 6-well plate for 48h, were then collected by trypsinization, and washed twice with PBS. Then, these cells were stained with annexin V-APC (APC) (NanJing KeyGen Biotech, Co., Ltd., China) and propidium iodide (PI) (NanJing KeyGen Biotech, Co., Ltd., China) following the protocol to measure cell apoptosis. At last, these samples were tested in the flow cytometer. This mean was able to identify the cells in early (APC+/PI-) and late (APC+/PI+) apoptosis.

### Drug Sensitivity Assay

Cells were cultured in 96 well plates at a density of 1 × 10^4^ cells/well overnight. Then, these cells were under the treatment of 5-Fluorouracil (5-Fu, final concentration of 0.1 mg/L) (APExBIO, United States) for 72h 33. Following, each well was added CCK-8 solution and then incubated at 37 ℃ for 4 h. Lastly, a microplate reader was applied to determine the absorbance.

Cells were cultured in 6 well plates at a density of 2 × 10^5^ cells/well overnight. Next, these cells underwent the disposal of 5-Fluorouracil (5-Fu, final concentration of 0.1 mg/L) for 72h. Then, cells were collected to evaluated cell apoptosis by flow cytometry as above description.

### Tumor formation in nude mice

Four-week-old male BALB/c nude mice were purchased from Hunan SJA Laboratory Animal Co., Ltd, and housed in specific-pathogen-free environment. All mice were handled based on standard-use protocols and animal welfare regulations. After one week accommodation, 5×10^5^ cells were injected subcutaneously into the left armpit of a BALB/c nude mouse. Tumor size was measured with a caliper. After 3 weeks of injection, mice were sacrificed, and subcutaneous tumors were excised. The tumor volume was calculated using the formula: long axis × (short axis) ^2^× 0.5. All animal procedures were approved by the Ethics Committee of Central South University.

### Statistical analysis

Data analysis was conducted with SPSS 23.0 and Graphpad Prism 6. Quantitative data were presented as mean ± SD values and analyzed with Student's test. The χ^2^ test and Fisher's exact test were applied to analyze the associations between APEX1 expression and clinicopathological characteristics. Survival analysis was performed with the Kaplan-Meier and Log-rank test. Univariate and multivariate analysis were performed with Cox's proportional hazards regression model. A *P* less than 0.05 was considered as statistical significance.

## Results

### Characteristic of patients

This study included 215 GBC patients which was comprised of 69 SC/ASC patients and 146 AC patients. The detail clinicopathological information of these patients was listed in Table 1.

### APEX1 is up-regulated in gallbladder cancer tissues

**Figure 1:**
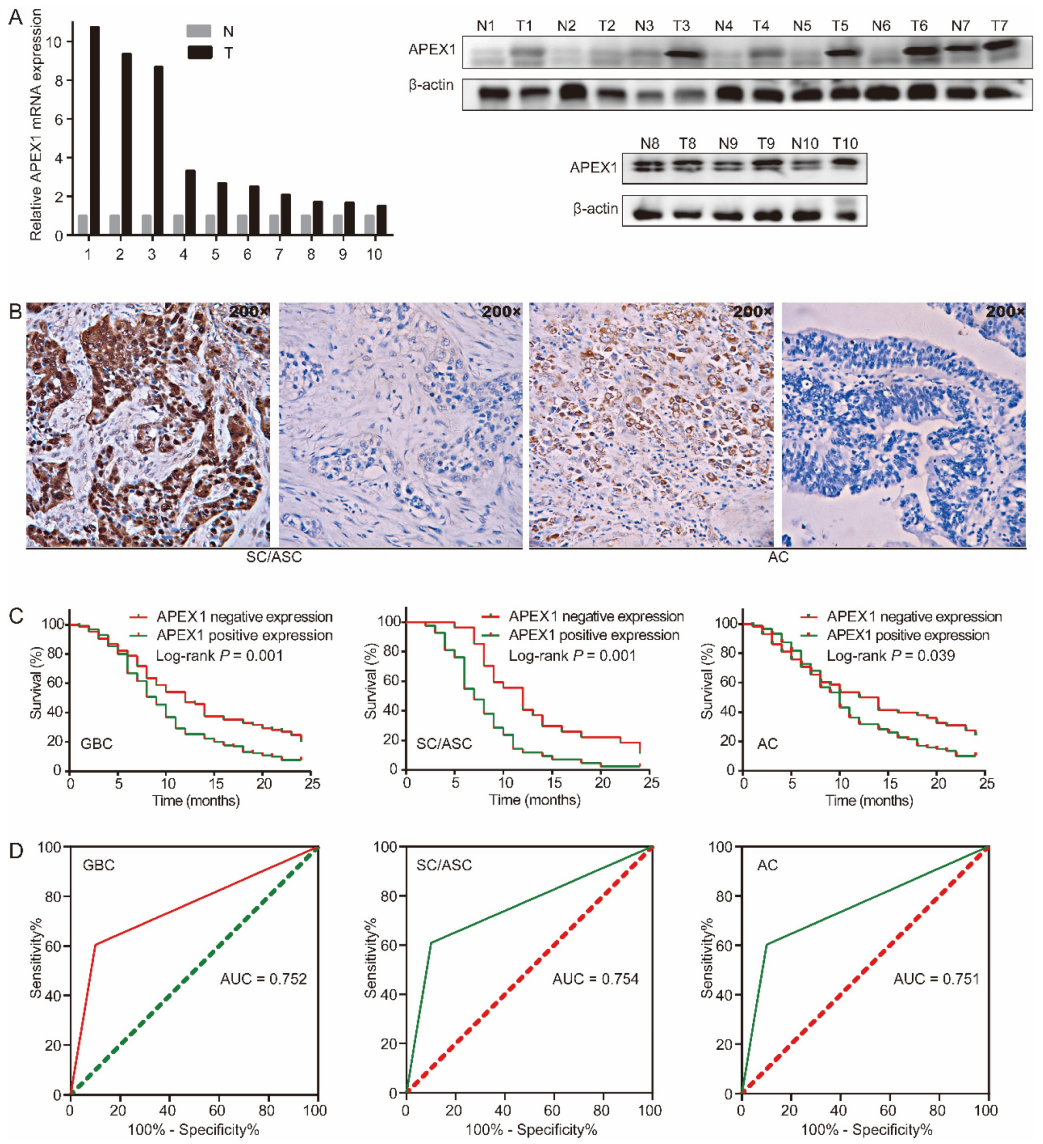
APEX1 expression is up-regulated in GBC and predicts poor prognosis. (A) APEX1 was overexpressed in human GBC tissues compared with corresponding adjacent normal tissues analyzed by qRT-PCR and western blot (T, GBC tissues; N, adjacent normal tissues). (B) Representative images of APEX1 positive and negative expression in gallbladder SC/ASC and AC. (C) The Kaplan-Meier curves of patients with positive or negative APEX1 expression in GBC, SC/ASC, and AC. (D) The ROC curves of APEX1 expression in GBC, SC/ASC, and AC.

We assessed APEX1 expression in GBC tissues and corresponding adjacent normal tissues by western blot and qRT-PCR. Compared with corresponding adjacent normal tissues, APEX1 mRNA and protein were significantly up-regulated in GBC tissues (Figure 1A). Then, APEX1 expression in 215 GBC tissues (including 146 ACs and 69 SC/ASCs) and 30 gallbladder epitheliums with chronic cholecystitis was detected by immunohistochemistry. The majority of positive APEX1 expression were localized in the cytoplasm of the SC/ASCs and ACs (Figure 1B). In comparison to gallbladder epitheliums with chronic cholecystitis, the positive rate of APEX1 expression in GBC including SC/ASC and AC was significantly higher (*P* < 0.001, Table 2). Furthermore, the epithelium of chronic cholecystitis with positive APEX1 expression presented moderate to severe dysplasia, indicated that APEX1 could be a biomarker to assess precancerous lesion in gallbladder.

### APEX1 positive expression is correlated with aggressive clinicopathological features and poor survival of GBC patients

We then analyzed the correlation between APEX1 expression and clinicopathological features in GBC patients (including SC/ASC and AC). The positive expression of APEX1 was significantly associated with lager tumor size (˃3cm), lymph node metastasis, locoregional invasion, advanced TNM stages (Ⅲ + Ⅳ), and only received biopsy (all *P* < 0.05, table 3) in GBC. Then, we further estimated the clinicopathological significance of APEX1 expression in different GBC subtypes. In gallbladder SC/ASC, APEX1 positive expression was significantly correlated to lager tumor size (˃3cm), lymph node metastasis, locoregional invasion, advanced TNM stages (Ⅲ + Ⅳ), and only received biopsy (all *P* < 0.05, table 3). In gallbladder AC, APEX1 positive expression was significantly related to a TNM stage of Ⅲ or Ⅳ (*P* = 0.030, Table 3).

Next, we evaluated the prognostic significance of APEX1 expression in GBC. In GBC, the patients with APEX1 positive expression had shorter mean survival time than those with APEX1 negative expression (10.346 vs 13.482 months, *P* = 0.001). In SC/ASC, the patients with APEX1 positive expression had shorter mean survival time than those with APEX1 negative expression (8.286 vs 13.333 months, *P* = 0.001). In AC, the patients with APEX1 positive expression had shorter mean survival time than those with APEX1 negative expression (11.330 vs 13.552 months, *P* = 0.039). Kaplan-Meier survival curves demonstrated that APEX1 positive expression was significantly associated with poor overall survival of GBC, SC/ASC, and AC (Figure 1C).

Moreover, univariate and multivariate cox regression analysis showed that positive APEX1 expression was an independent risk factor for overall survival of GBC patients including SC/ASC and AC (Table 4 and Table 5). Furthermore, the receiver operating characteristic (ROC) curve revealed that APEX1 presented clinicopathological diagnostic efficacy in GBC, SC/ASC, and AC. The AUC of APEX1 expression in GBC, SC/ASC, and AC was 0.752 (95%CI: 0.673-0.832), 0.754 (95%CI: 0.656-0.853), and 0.751 (95%CI: 0.667-0.836), respectively (Figure 1D). Above results demonstrated that APEX1 might be a novel potential diagnostic and prognostic biomarker of GBC.

### APEX1 is overexpressed in CD133^+^ GBC-SD cell compared with normal GBC-SD cell

**Figure 2:**
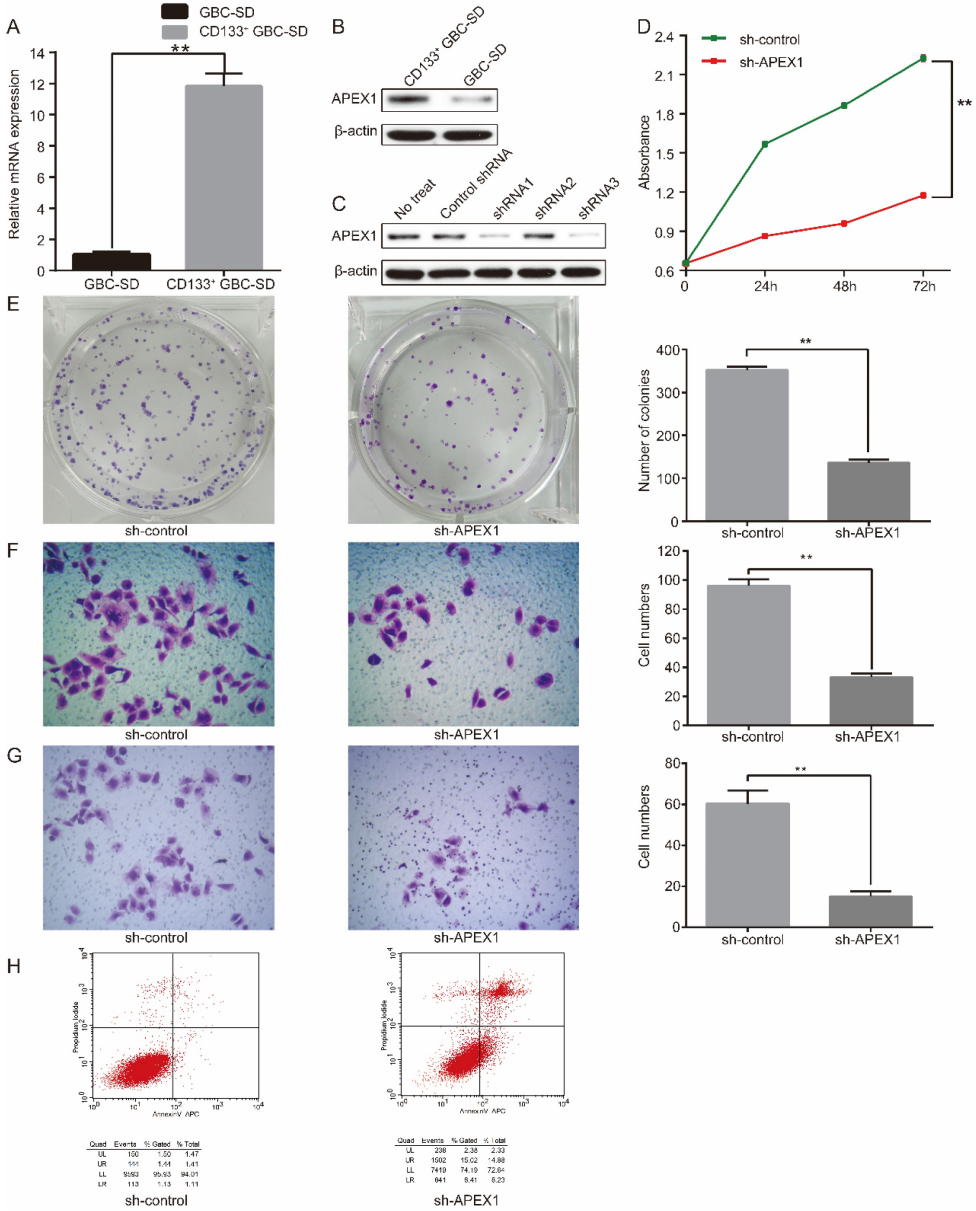
APEX1 knockdown suppresses the proliferation, migration and invasion of CD133^+^ GBC-SD cells, and promotes CD133^+^ GBC-SD cells apoptosis. (A) mRNA and (B) protein expressions of APEX1 in CD133^+^ GBC-SD cells and GBC-SD cells were detected by qRT- PCR and western blot. (C) APEX1 protein expression was tested via western blot in CD133^+^ GBC-SD cells transfected with different APEX1 shRNAs. (D) CCK8 and (E) colony formation assays were applied to examine the proliferation of CD133^+^ GBC-SD cells transfected with control shRNA or APEX1 shRNA. (F) and (G) Transwell assay was subjected to measure the migration and invasion of CD133^+^ GBC-SD cells transfected with control shRNA or APEX1 shRNA. (H) Apoptosis of CD133^+^ GBC-SD cells transfected with control shRNA or APEX1 shRNA was tested via flow cytometry assay; UL: necrosis cells, UR: late apoptosis cells, LL: normal cells, LR: early apoptosis cells. ** *P*<0.01.

To further explore the cellular biological role of APEX1 in GBC, the expression of APEX1 was detected in CD133^+^ GBC-SD cell and normal GBC-SD cell by western blot and qRT-PCR. Compared with GBC-SD cells, APEX1 was overexpressed in CD133^+^ GBC-SD cells both in mRNA and protein levels (Figure 2A and 2B).

### APEX1 promotes CD133^+^ GBC-SD cell proliferation, migration, invasion, and resistance to 5-Fu, and inhibits CD133^+^ GBC-SD cell apoptosis *in vitro*

To further assess the biological function of APEX1, short hairpin RNA (shRNA) targeting APEX1 was introduced into CD133^+^ GBC-SD cells. Three shRNAs (shRNA1, shRNA2, shRNA3) were framed to knockdown APEX1 in CD133^+^ GBC-SD cells named as CD133^+^ GBC-SD-shAPEX1 subsequently. To evaluate the efficacy of shRNA knockdown, western blot was performed to identify the expression of APEX1. Among the three shRNAs, shRNA3 was the most effective one and was chosen for further experiments (Figure 2C).

The CCK8 assay was applied to investigate the proliferation capacity. CD133^+^-GBC-SD-shAPEX1 presented a lower absorbance than CD133^+^-GBC-SD- shcontrol, which suggested a lower proliferation (Figure 2D). Consistently, colony formation assay showed that CD133^+^-GBC-SD-shAPEX1 formed less colonies compared with CD133^+^-GBC-SD- shcontrol (Figure 2E). Migration and invasion capacity was tested via transwell assay. Results showed that CD133^+^-GBC-SD-shAPEX1 had less migration cells and invasion cells than CD133^+^-GBC-SD-shcontrol (Figure 2F and 2G). Flow cytometry was used to determine the apoptosis capacity, and CD133^+^-GBC-SD-shAPEX1 presented a higher apoptosis rate than CD133^+^-GBC-SD-shcontrol (Figure 2H). These results demonstrated that APEX1 promotes CD133^+^-GBC-SD cell proliferation, migration, and invasion ability, and inhibits CD133^+^-GBC-SD cell apoptosis capacity *in vitro*.

**Figure 3:**
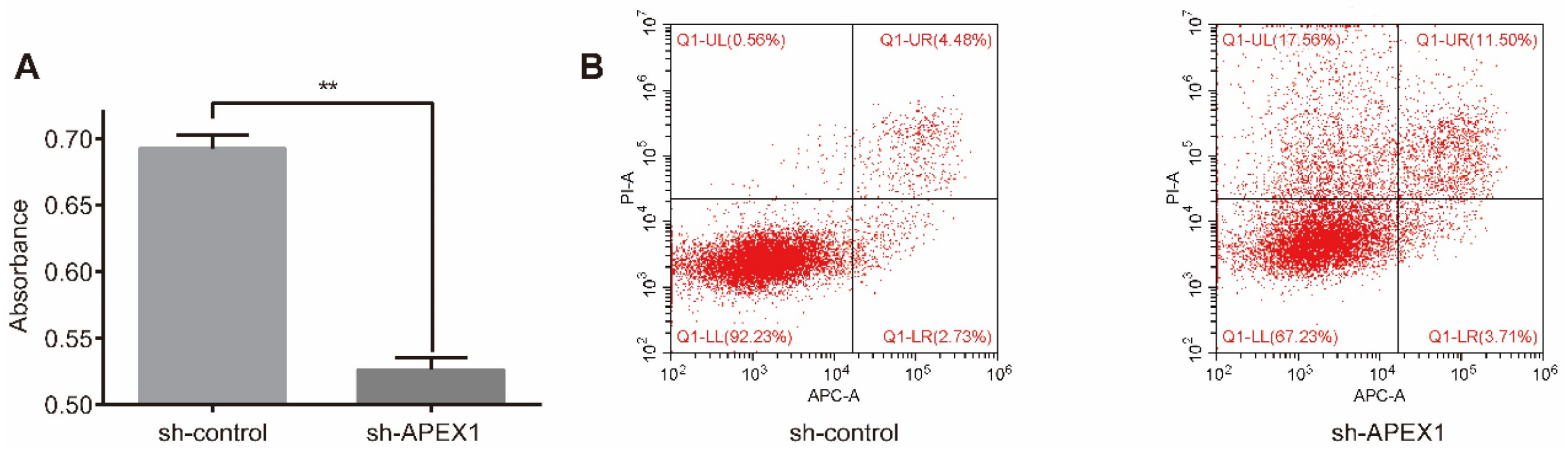
APEX1 knockdown increased the sensitivity of CD133^+^ GBC-SD cells to 5-Fu. (A) After treatment with 5-Fu for 72 h, cell viability of CD133^+^ GBC-SD-shAPEX1 cells and CD133^+^ GBC-SD-shcontrol cells was tested via CCK8. (B) After treatment with 5-Fu for 72 h, apoptosis of CD133^+^ GBC-SD-shAPEX1 cells and CD133^+^ GBC-SD-shcontrol cells was assessed by flow cytometry assay; Q1-UL: necrosis cells, Q1-UR: late apoptosis cells, Q1-LL: normal cells, Q1-LR: early apoptosis cells.

To explore the effect of APEX1 on chemo-resistance, CD133^+^-GBC-SD-shAPEX1 was treated with 5-Fu for 72h, and then CCK8 assay and flow cytometry assay were performed. CCK8 assay showed that CD133^+^-GBC-SD-shAPEX1 presented a lower absorbance than CD133^+^-GBC-SD-shcontrol (Figure 3A), which indicated that APEX1 could reduce the destruction of 5-Fu to CD133^+^-GBC-SD cells. Flow cytometry assay presented the apoptosis rate and necrosis rate of CD133^+^-GBC-SD-shAPEX1 were higher than CD133^+^-GBC-SD-shcontrol (Figure 3B). These results suggested that APEX1 facilitates CD133^+^-GBC-SD cell resistance to 5-Fu via inhibiting cell apoptosis and necrosis *in vitro*.

### APEX1 regulates Notch signaling pathway in CD133^+^ GBC-SD cell

**Figure 4:**
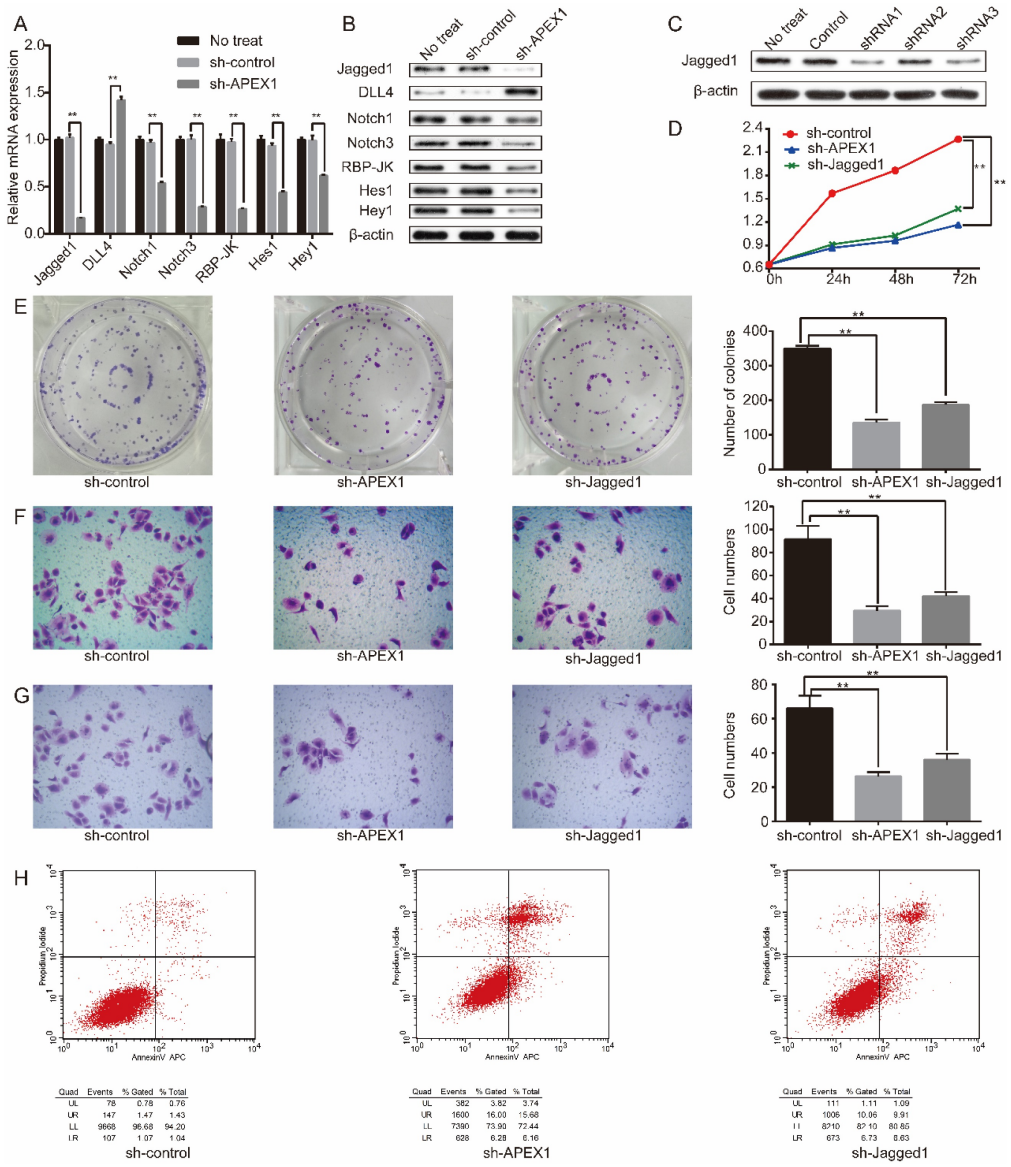
APEX1 can regulate Notch signal pathway including Jagged1, and APEX1 knockdown and Jagged1 knockdown present similar biological function in regulating proliferation, migration, invasion, and apoptosis of CD133^+^ GBC-SD cells. (A) qRT-PCR and (B) western blot were used to evaluated the expression of Notch signaling members. (C) Jagged1 protein expression was detected by western blot in CD133^+^ GBC-SD cells transfected with different Jagged1 shRNAs. (D) CCK8 and (E) colony formation assays were used to assess the proliferation of CD133^+^ GBC-SD cells transfected with control shRNA, APEX1 shRNA, or Jagged1 shRNA. (F) and (G) Transwell assay was subjected to tested the migration and invasion of CD133^+^ GBC-SD cells transfected with shRNA, APEX1 shRNA, or Jagged1 shRNA. (H) Apoptosis of CD133^+^ GBC-SD cells transfected with control shRNA, APEX1 shRNA, or Jagged1 shRNA was tested via flow cytometry assay; UL: necrosis cells, UR: late apoptosis cells, LL: normal cells, LR: early apoptosis cells. ** *P*<0.01.

Then, we explored the potential molecular mechanism of APEX1 in regulating biological function of CD133^+^-GBC-SD cells. Previous studies revealed that Notch signaling was associated with GBC tumorigenesis 34, 35. Thus, we further verified the correlation between APEX1 and Notch signaling pathway. After APEX1 knockdown in CD133^+^ GBC-SD cells, we detected the expression of Notch signaling members, including Notch1, Notch3, Jagged1, DLL4, Hes1, Hey1, and RBP-JK expression. Results showed that APEX1 knockdown in CD133^+^ GBC-SD cells significantly increased DLL4 expression, and obviously decreased the expression of Notch1, Notch3, Jagged1, Hes1, Hey1, and RBP-JK both in mRNA and protein levels (Figure 4A and 4B), suggesting that APEX1 was capable to regulate Notch signaling pathway in CD133^+^ GBC-SD cells. Considering that APEX1 could drive colon cancer progression via upregulating Jagged1 26, we speculated that APEX1 might modulate CD133^+^ GBC-SD cell biological function by regulating Jagged1.

### APEX1 and Jagged1 have similar biological role in CD133^+^ GBC-SD cell

To further compared the biological role between APEX1 and Jagged1, APEX1 and Jagged1 expression was manipulated by short hairpin RNA (shRNA) knockdown in CD133^+^ GBC-SD cells, respectively. Three shRNAs (shRNA1, shRNA2, shRNA3) were constructed to knockdown Jagged1 in CD133^+^ GBC-SD cells named as CD133^+^ GBC-SD-shJagged1 subsequently. To evaluate the efficacy of shRNA knockdown, western blot was conducted to examine the expression of Jagged1, and the most effective one was shRNA1 which was selected for further study (Figure 4C).

CCK8 assay showed that the absorbance of CD133^+^ GBC-SD-shcontrol was significantly higher than CD133^+^ GBC-SD-shAPEX1 and CD133^+^ GBC-SD-shJagged1 (Figure 4D). In colony assay, the number of cell colonies of CD133^+^ GBC-SD-shAPEX1 and CD133^+^ GBC-SD-shJagged1 apparently reduced in comparison of CD133^+^ GBC-SD-shcontrol (Figure 4E). In transwell assay, migration and invasion cells in CD133^+^ GBC-SD-shcontrol were much more than CD133^+^ GBC-SD-shAPEX1 and CD133^+^ GBC-SD-shJagged1 (Figure 4F and 4G). Flow cytometry assay presented that the apoptosis rate of CD133^+^ GBC-SD-shAPEX1 and CD133^+^ GBC-SD-shJagged1 obviously enhanced compared with CD133^+^ GBC-SD-shcontrol (Figure 4H). These results demonstrated that Jagged1 and APEX1 had similar biological function in CD133^+^ GBC-SD cells that they could promote cell proliferation, migration, and invasion, and suppress cell apoptosis *in vitro*.

### APEX1 promotes CD133^+^ GBC-SD cell proliferation, invasion, and migration, and inhibits CD133^+^ GBC-SD cell apoptosis *in vitro* by Jagged1

**Figure 5:**
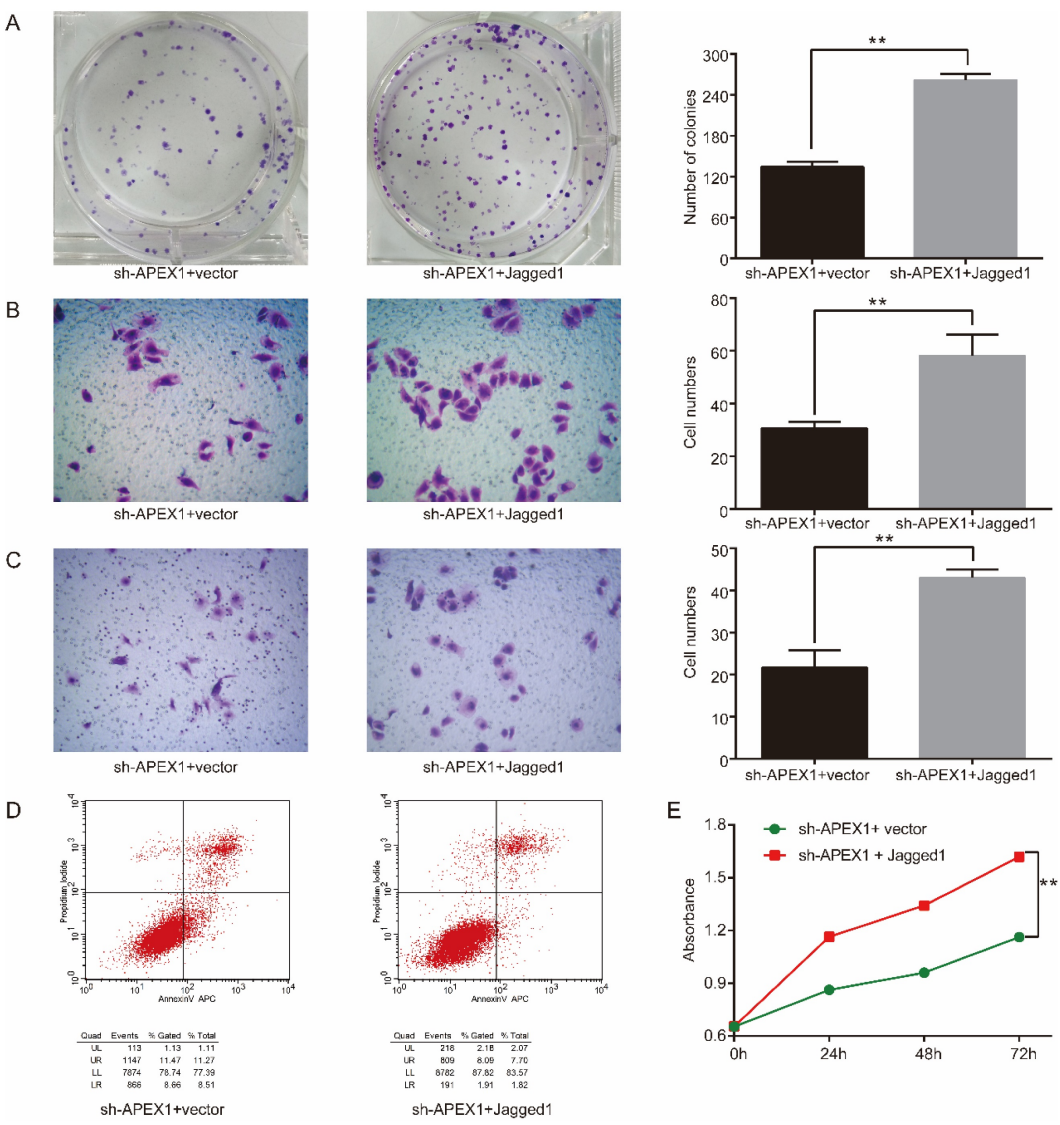
Jagged1 overexpression facilitates tumorigenicity in APEX1-knockdown CD133^+^ GBC-SD cells. (A) Colony formation assays were used to assess the proliferation of CD133^+^ GBC-SD-shAPEX1 cells transfected with control vector or Jagged1 expression vector. (B) and (C) Transwell assays were subjected to tested the migration and invasion of CD133^+^ GBC-SD-shAPEX1 cells transfected with control vector or Jagged1 expression vector. (D) Apoptosis of CD133^+^ GBC-SD-shAPEX1 cells transfected with control vector or Jagged1 expression vector was tested via flow cytometry assay; UL: necrosis cells, UR: late apoptosis cells, LL: normal cells, LR: early apoptosis cells. (E) CCK8 assays were used to assess the proliferation of CD133^+^ GBC-SD-shAPEX1 cells transfected with control vector or Jagged1 expression vector. ** *P*<0.01.

Considering that APEX1 could upregulate Jagged1 expression and they possessed similar biological function in CD133^+^ GBC-SD cells, we speculated that APEX1 may modulate the biological behavior of CD133^+^ GBC-SD cells via upregulating Jagged1 expression. To further verify this hypothesis, Jagged1 ectopic expression plasmid was transfected into CD133^+^ GBC-SD-shAPEX1 cells. Overexpression of Jagged1 in CD133^+^ GBC-SD-shAPEX1 cells dramatically enhanced cell proliferation, clonogenicity, migration, and invasion capacity, and markedly suppressed cell apoptosis capacity compared with the vector-transfected CD133^+^ GBC-SD-shAPEX1 cells (Figure 5), which suggested that overexpression of Jagged1 in CD133^+^ GBC-SD cells was able to rescue the biological behavior variation induced by APEX1 knockdown. These results indicated that APEX1 promotes cell proliferation, invasion, and migration, and inhibits cell apoptosis by Jagged1 in CD133^+^ GBC-SD cells.

### APEX1 promotes CD 133^+^ GBC-SD cell growth *in vivo* by Jagged1

**Figure 6:**
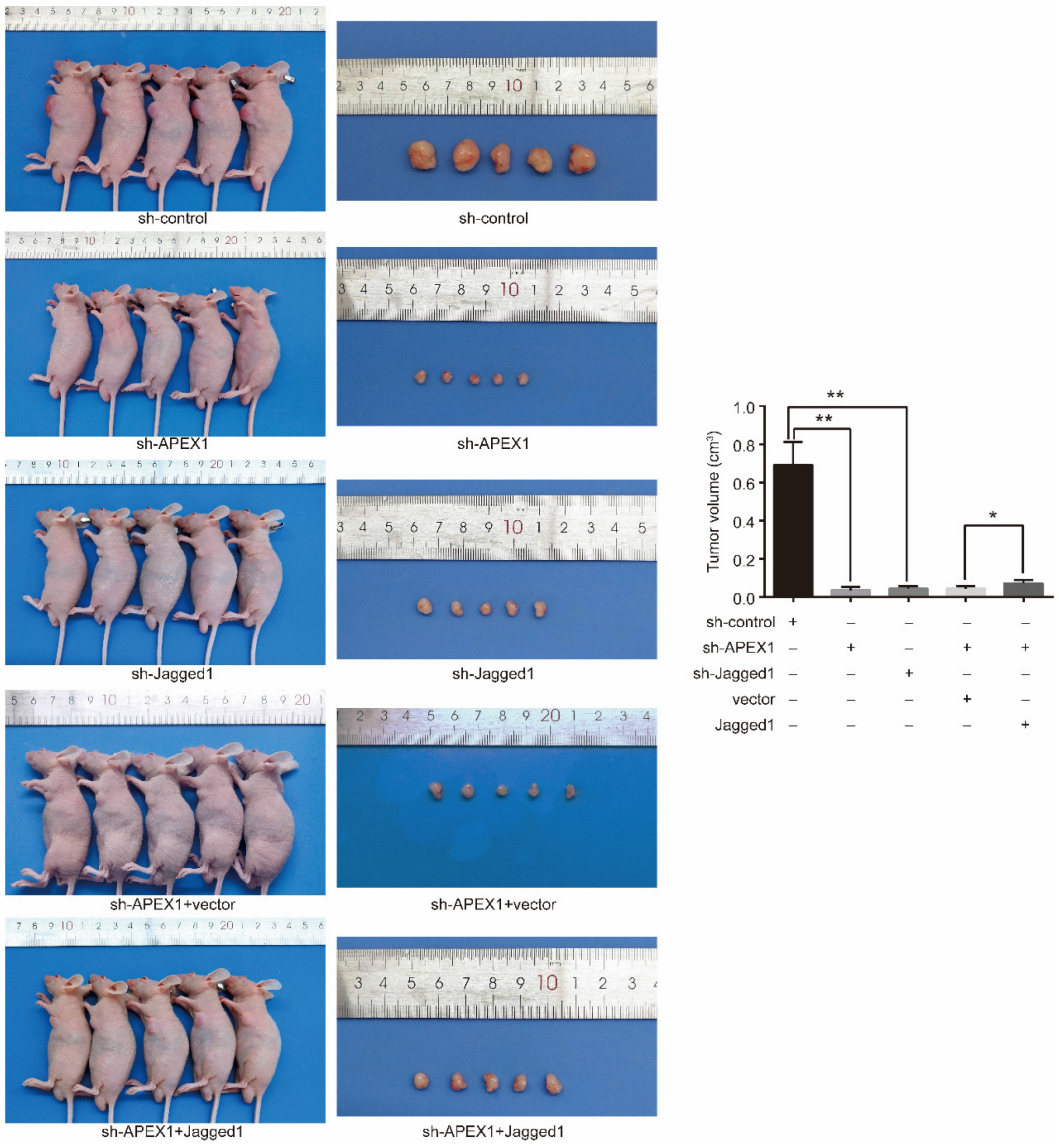
APEX1 modulates tumor growth via Jagged1 *in vivo*. Tumors formed by CD133^+^ GBC-SD-shAPEX1 cells and CD133^+^ GBC-SD-shJagged1 cells were obviously smaller than CD133^+^ GBC-SD-shcontrol cells. Tumors formed by Jagged1-transfected CD133^+^ GBC-SD-shAPEX1 cells were remarkably larger than vector-transfected CD133^+^ GBC-SD-shAPEX1 cells. * *P*<0.05, ** *P*<0.01.

Next, we investigated the role of APEX1 and Jagged1 in tumor formation *in vivo* applying mouse xenograft models. Firstly, the models were constructed via subcutaneously implanting CD133^+^ GBC-SD-shAPEX1 cells and CD133^+^ GBC-SD-shJagged1 cells into the left armpit of nude mice. After 3 weeks, CD133^+^ GBC-SD -shcontrol cell-derived tumors at the subcutaneously implanting location were lager and grew more rapidly than CD133^+^ GBC-SD-shAPEX1 cell-derived tumors and CD133^+^ GBC-SD-shJagged1 cells-derived tumors (Figure 6), indicating that knockdown of APEX1 or Jagged1 in CD133^+^ GBC-SD cells suppressed the tumor growth *in vivo*.

To further identify whether APEX1 affected the tumor growth *in vivo* via Jagged1, Jagged1-transfected CD133^+^ GBC-SD-shAPEX1 cells or vector-transfected CD133^+^ GBC-SD-shAPEX1 cells were subcutaneously injected into the left armpit of nude mice. After 3 weeks, the mice injected Jagged1-transfected CD133^+^ GBC-SD-shAPEX1 cells presented lager tumors at the subcutaneously implanting location compared with the mice injected vector-transfected CD133^+^ GBC-SD-shAPEX1 cells (Figure 6). These results suggested that the reduced tumor growth *in vivo* caused by APEX1 knockdown could be restored by Jagged1 ectopic expression in CD133^+^ GBC-SD-shAPEX1 cells. Based on the above results, this experiment demonstrated that APEX1 promote CD133^+^ GBC-SD cell growth *in vivo* by Jagged1.

## Discussion

As the most common malignant of the biliary duct system, GBC exhibits aggressive clinicopathological characteristics and poor prognosis. Currently, there are no appropriate biological makers for diagnosing GBC in early stage. In this study, we found that APEX1 was overexpressed in GBC and correlated to adverse clinicopathological characteristic and poor prognosis of GBC patients. APEX1 was an independent risk prognostic factor for GBC, and presented clinicopathological diagnostic efficacy in GBC, indicating that APEX1 might be a novel potential biological maker for diagnosis and prognosis for GBC. Moreover, the present study demonstrated that APEX1 facilitated cell growth, invasion, and migration, and suppressed cell apoptosis via regulating Jagged1 in CD133^+^ GBC-SD cells. Therefore, APEX1 might function an important role in GBC tumorigenesis.

As an important multifunctional protein, APEX1 plays a vital role in the BER pathway. APEX1 possesses the function of modulating transcription factors, which is able to impact the combination of tumorigenesis associated transcription factors to DNA, such as AP1, NF-κB, and p53 23. A variety of studies revealed that APEX1 is up-regulated in many human solid cancer tissues including melanoma, glioma, prostate cancer, ovarian cancer, bladder cancer, osteosarcoma, and liver cancer 23, 28-30. Similarly, this study confirmed that APEX1 expression in GBC was significantly higher than that in corresponding adjacent normal tissues and chronic cholecystitis tissues. The chronic cholecystitis tissues with APEX1 positive expression occurred moderate to severe dysplasia, suggesting that APEX1 may be involved in the evolution of benign lesions into GBC. APEX1 positive expression is associated with poor clinicopathological features and adverse prognosis of several human cancer, such as prostate cancer and osteosarcoma 28, 30. Consistently, we found that the GBC patients with APEX1 positive expression showed aggressive clinicopathological characteristic and poor outcome. Moreover, this study revealed that APEX1 positive expression was an independent risk factors for GBC, which was similar to the previous founding in prostate cancer 28. Kim et, al have identified that APEX1 expression is a potential diagnostic biological maker of clear cell renal, liver cancer, and cholangiocarcinoma 25. Likewise, the ROC curve showed that APEX1 positive expression exhibited predictive value for GBC diagnosis in this study.

To our knowledge, this study firstly revealed the biological role of APEX1 in GBC cells. CD133^+^ tumor cell is a subtype of tumor cells with some property of stem cells. CD133^+^/ESA^+^ colon cancer stem cells present significantly higher APEX1 mRNA expression than corresponding colon cancer cells 27. Consistently, both protein and mRNA expression of APEX1 in CD133^+^ GBC-SD cells was obviously higher than in GBC-SD cells, indicating that CD133^+^ GBC-SD cells may possess better capacity to repair damaged DNA. Previous studies have reported that CD133^+^ tumor cells present stronger DNA repairing ability than CD133^-^ tumor cells in glioma, medulloblastoma, prostate cancer and lung cancer 15-18. The enhance of DNA repairing ability of cancer cells is closely associated with chemoresistance. As a key rate-limiting enzyme in DNA BER pathway, APEX1 functions an irreplaceable role in tumor chemotherapy resistance. For example, APEX1 promotes resistance of biliary cancer cell lines to 5-Fu, cisplatin, and gemcitabine, and chemosensitive cases show lower APEX1 expression compared with chemoresistant cases in biliary cancer 36. Moreover, inhibiting APEX1 expression can enhance the sensitive of colon cancer cells to 5-Fu 27. This study also demonstrated that knockdown APEX1 could improve the responsive of CD133^+^ GBC-SD cells to 5-Fu via promoting cell necrosis and apoptosis. Thus, we supposed that APEX1 may be a potential novel target to increase chemosensitivity of GBC.

APEX1 is involved in the progression of some human cancer types. In this study, we found that GBC patients with APEX1 positive expression presented a lager tumor size, and down-regulating APEX1 in CD133^+^ GBC-SD cells was capable of suppressing tumor growth *in vivo*. Tumor size is mainly determined by cancer cell proliferation and apoptosis capacity. Previous studies have identified that APEX1 can facilitate cell proliferation and restrain cell apoptosis in pancreatic cancer cells, ovarian cancer cells, and hepatic cancer cells 24, 29, 37. In agreement with previous reports, this study demonstrated that APEX1 knockdown in CD133^+^ GBC-SD cells significantly decreased cell proliferation capacity and increased cell apoptosis ability *in vitro*, suggesting that APEX1 may promote GBC growth via regulating cell proliferation and apoptosis. Activating invasion and metastasis is a hallmark of cancer. In the present study, the GBC patients with APEX1 positive expression was more likely to occur lymph node metastasis and surrounding organs and tissues invasion, suggesting that APEX1 may promote GBC metastasis. Migration and invasion are the necessary prelude to tumor metastasis. APEX1 functions a part in stimulating migration and invasion of several human cancer types including colon cancer cells, pancreatic cancer cells, and lung cancer cells 26, 38, 39. Similarly, this study showed that APEX1 knockdown significantly restrained CD133^+^ GBC-SD cells migration and invasion in transwell assays, demonstrating that APEX1 facilitates GBC cells invasion and migration. Thus, these above results confirmed that APEX1 promoted GBC progression.

As a crucial member of Notch signal pathway, Jagged1 plays an important role in both physiological and pathological conditions 40. Jagged1 is participated in occurrence of human cancers, and abnormal expression of Jagged1 can lead to alterations in biological behaviors of tumor cells, such as colon cancer cells, pancreatic cancer cells, and ovarian carcinoma cells 41-43. As far as we know, the biological function of Jagged1 in CD133^+^ GBC cells was firstly illustrated in this study. Consistent with previous studies, Jagged1 knockdown caused significant variations in proliferation, migration, invasion, and apoptosis of CD133^+^ GBC-SD cells. A previous finding has showed that Jagged1 positive expression is related to lager tumor size, lymph node metastasis, and invasion of GBC 34, which was further confirmed in cell functional experiments in this study.

APEX1 can activate Notch signal pathway via Jagged1 in colon cancer 26. APEX1 knockdown in cholangiocarcinoma cells is able to down-regulate Jagged1 expression 36. Likewise, we found that APEX1 could regulate Notch signal pathway including Jagged1 in CD133^+^ GBC-SD cells, which was never reported. Furthermore, both APEX1 knockdown and Jagged1 knockdown inhibited CD133^+^ GBC-SD cells proliferation, migration, invasion, and tumor growth, and promoted CD133^+^ GBC-SD cells apoptosis, demonstrating that APEX1 and Jagged1 possessed similar biological function in CD133^+^ GBC-SD cells. Moreover, a previous study has confirmed that APEX1 can facilitate colon cancer oncogenesis by regulating Jagged1 expression 26. Therefore, we hypothesized that APEX1 might function biological roles in CD133^+^ GBC-SD cells via modulating Jagged1. To further explore the relationship between APEX1 and Jagged1, the rescue experiments were performed in this study. As we expected, Jagged1 overexpression could recover tumorigenic phenotypes in APEX1 knockdown CD133^+^ GBC-SD cells, including proliferation, migration, invasion, apoptosis, and tumor growth. Thus, our data verified that APEX1 affected biological features of CD133^+^ GBC-SD cells by regulating Jagged1 expression.

In conclusion, the present study showed that APEX1 was an independent poor prognostic marker and presented diagnostic efficacy in GBC. Additionally, APEX1 played an essential role in facilitating proliferation, migration, invasion, and tumor growth, and inhibiting apoptosis by modulating Jagged1 in CD133^+^ GBC-SD cells. Thus, this study indicated that APEX1 may be a promising prognostic and diagnostic biomarker, and a potential therapeutic target for GBC.

## Figures and Tables

**Table 1:** Comparison of gallbladder SC/ASC and AC clinicopathological features including APEX1 expression status.

Clinicopathological characteristics	Number of GBC (%)	Number of SC/ASC (%)	Number of AC (%)
Gender			
Male	86 (40.0)	25 (36.2)	61 (41.8)
Female	129 (60.0)	44 (63.8)	85 (58.2)
Age			
≤45 years	23 (10.7)	3 (4.3)	20 (13.7)
>45 years	192 (89.3)	66 (95.7)	126 (86.3)
Differentiation			
Well	70 (32.5)	19 (27.5)	51 (34.9)
Moderate	87 (40.5)	33 (47.8)	54 (37.0)
Poor	58 (27.0)	17 (24.6)	41 (28.1)
Maximum tumor diameter			
≤3 cm	120 (55.8)	30 (43.5)	90 (61.6)
>3 cm	95 (44.2)	39 (56.5)	56 (38.4)
Gallstone			
No	109 (50.7)	31 (44.9)	78 (53.4)
Yes	106 (49.3)	38 (55.1)	68 (46.6)
TNM stages			
I + II	106 (49.3)	29 (42.0)	77 (52.7)
III + IV	109 (50.7)	40 (58.0)	69 (47.3)
Lymph node metastasis			
No	107 (49.8)	27 (39.1)	80 (54.8)
Yes	108 (50.2)	42 (60.9)	66 (45.2)
Locoregional invasion			
No	96 (44.7)	24 (34.8)	72 (49.3)
Yes	119 (55.3)	45 (65.2)	74 (50.7)
Surgical methods			
Radical	102 (47.4)	27(39.1)	75 (51.4)
Palliative	78 (36.3)	28 (40.6)	50 (34.2)
Without resection	35 (16.3)	14 (20.3)	21 (14.4)
APEX1			
-	85 (39.5)	27(39.1)	58 (39.7)
+	130 (60.5)	42 (60.9)	88 (60.3)

**Table 2:** Comparison of APEX1 expression in GBC and gallbladder epitheliums with chronic cholecystitis.

Tissue types	APEX1 positive expression (%)	χ^2^	*P*
GBC	130 (60.5)	27.017	0.000
SC/ASC	42 (60.9)	21.823	0.000
AC	88 (60.3)	25.189	0.000
Gallbladder epitheliums with chronic cholecystitis	3 (10)		

**Table 3:** Correlations of APEX1 protein expression with the clinicopathological characteristics of gallbladder cancer.

Clinicopathological characteristics	GBC		SC/ASC		AC	
	Positive Number (%)	*P*	Positive Number (%)	*P*	Positive Number (%)	*P*
Differentiation						
Well	40 (57.1)	0.090	10 (52.6)	0.297	30 (58.8)	0.236
Moderately	48 (55.2)		19 (57.6)		29 (53.7)	
Poorly	42 (72.4)		13 (76.5)		29 (70.7)	
Tumor size						
≤3cm	63 (52.5)	0.007	12 (40.0)	0.002	51 (56.7)	0.259
˃3cm	67 (70.5)		30 (76.9)		37 (66.1)	
Gallstone						
No	73 (67.0)	0.048	22 (71.0)	0.121	51 (65.4)	0.177
Yes	57 (53.8)		20 (52.6)		37 (54.4)	
Lymph node metastasis						
No	56(52.3)	0.015	11 (40.7)	0.006	45 (56.3)	0.274
Yes	74 (68.5)		31 (73.8)		43 (65.2)	
Invasion						
No	48 (50.0)	0.005	9 (37.5)	0.004	39 (54.2)	0.137
Yes	82 (68.9)		33 (73.3)		49 (66.2)	
TNM stage						
Ⅰ + Ⅱ	53 (50.0)	0.002	13 (44.8)	0.020	40 (51.9)	0.030
Ⅲ + Ⅳ	77(70.6)		29 (72.5)		48 (69.6)	
Surgery						
Radical	52(51.0)	0.019	10 (37.0)	0.005	42 (56.0)	0.422
Palliative	52(66.7)		21 (75.0)		31 (62.0)	
Biopsy	26(74.3)		11 (78.6)		15 (71.4)	

**Table 4:** Univariate Cox regression analysis of survival rate in GBC, SC/ASC and AC patients.

Groups	Factors	GBC		SC/ASC		AC	
		*P*	HR (95%CI)	*P*	HR (95% CI)	*P*	HR (95% CI)
Differentiated degree	Well/moderately/Poorly	0.000	2.173(1.770-2.667)	0.000	2.040(1.394-2.983)	0.000	2.227(1.740-2.851)
Tumor size	≤3 cm/>3 cm	0.034	1.369(1.024-1.830)	0.034	1.765(1.044-2.984)	0.000	2.331(1.614-3.367)
Gallstone	No/Yes	0.126	1.252(0.939-1.670)	0.088	1.565(0.935-2.261)	0.981	1.004(0.704-1.433)
TNM stage	I+II/III+IV	0.000	6.069(4.298-8.569)	0.000	6.830(3.619-12.890)	0.000	5.923(3.898-9.002)
Lymph node metastasis	No/Yes	0.000	4.973(3.529-7.008)	0.000	4.550(2.453-8.438)	0.000	5.021(3.312-7.612)
Invasion	No/Yes	0.000	9.007(6.115-13.268)	0.000	5.453(2.942-10.104)	0.000	12.808(7.412-22.131)
Surgery	Radical/ Palliative/ Biopsy	0.000	5.012(3.862-6.505)	0.000	4.240(2.709-6.637)	0.000	5.693(4.081-7.940)
APEX1	-/+	0.002	1.602(1.185-2.165)	0.002	2.268(1.349-3.815)	0.013	1.610(1.107-2.340)

Abbreviation: HR, hazard risk ratio; CI, confidence interval; -, negative expression; +, positive expression.

**Table 5:** Multivariate Cox regression analysis of survival rate in GBC, SC/ASC and AC patients.

Groups	Factors	GBC		SC/ASC		AC	
		*P*	HR (95%CI)	*P*	HR (95% CI)	*P*	HR (95% CI)
Differentiated degree	Well/moderately/Poorly	0.000	1.508(1.209-1.882)	0.001	1.970(1.308-2.967)	0.002	1.551(1.179-2.041)
Tumor size	≤3 cm/>3 cm	0.031	1.353(1.028-1.780)	0.030	1.808(1.059-3.087)	0.012	1.790(1.138-2.814)
Gallstone	No/Yes	0.103	1.275(0.952-1.708)	0.332	1.328(0.748-2.359)	0.488	1.142(0.784-1.664)
TNM stage	I+II/III+IV	0.020	1.893(1.106-3.238)	0.002	3.219(1.534-6.752)	0.001	3.127(1.553-6.295)
Lymph node metastasis	No/Yes	0.001	2.217(1.361-3.611)	0.004	3.522(1.501-8.261)	0.000	3.811(2.040-7.122)
Invasion	No/Yes	0.000	3.947(2.395-6.506)	0.012	4.080(1.359-12.250)	0.000	6.666(3.344-13.289)
Surgery	Radical/ Palliative/Biopsy	0.000	2.467(1.821-3.343)	0.003	2.349(1.336-4.131)	0.000	2.354(1.566-3.538)
APEX1	-/+	0.004	1.590(1.155-2.189)	0.006	2.284(1.274-4.096)	0.032	1.575(1.039-2.386)

Abbreviation: HR, hazard risk ratio; CI, confidence interval; -, negative expression; +, positive expression.

## Abbreviations

GBC: gallbladder cancer
AC: adenocarcinoma
SC/ASC: squamous cell/adenosquamous carcinoma
APEX1: Apurinic-apyrimidinic endonuclease-1
EMT: epithelial-mesenchymal transition
BER: base excision repair
EGR1: Early Growth Response 1
NF-κB: nuclear factor kappa B
HIF-1α: hypoxia inducible factor-1α
DLL4: Delta-like ligand 4
RBP-JK: recombination signal binding protein for JK
Hes1: hairy and enhancer of split 1
Hey1: Hes Related Family BHLH Transcription Factor With YRPW Motif 1
CCK8: Cell Counting Kit-8
5-FU: 5-Fluorouracil
ROC: receiver operating characteristic
AUC: Area Under Curve
shRNA: short hairpin RNA
TNM: tumor-node- metastasis
